# Silencing of Pokemon Enhances Caspase-Dependent Apoptosis via Fas- and Mitochondria-Mediated Pathways in Hepatocellular Carcinoma Cells

**DOI:** 10.1371/journal.pone.0068981

**Published:** 2013-07-17

**Authors:** Yu-Qin Zhang, Chuan-Xing Xiao, Bi-Yun Lin, Ying Shi, Yun-Peng Liu, Jing-Jing Liu, Bayasi Guleng, Jian-Lin Ren

**Affiliations:** 1 Department of Gastroenterology, Zhongshan Hospital affiliated to Xiamen University, Xiamen, China; 2 Faculty of Clinical Medicine, Medical College of Xiamen University, Xiamen, China; The University of Hong Kong, China

## Abstract

The role of Pokemon (POK erythroid myeloid ontogenic actor), a recently identified POK transcription factor with proto-oncogenic activity, in hepatocellular carcinogenesis has only been assessed by a few studies. Our previous study revealed that Pokemon is overexpressed in hepatocellular carcinomas (HCC) and promotes HCC cell proliferation and migration via an AKT- and ERK- dependent manner. In the present study, we used the TUNEL assay and FACS analysis to demonstrate that oxaliplatin induced apoptosis was significantly increased in cells with silenced Pokemon. Western blots showed that p53 expression and phosphorylation were significantly increased in Pokemon defective cells, thereby initiating the mitochondria-mediated and death receptor-mediated apoptotic pathways. In the mitochondria-mediated pathway, expression of pro-apoptotic Bcl-2 family members (including Bad, Bid, Bim and Puma) as well as AIF was increased and decreasing the mitochondrial membrane potential resulted in cytochrome C released from mitochondrial in HepG2 si-Pokemon cells. In addition, upon oxaliplatin treatment of Pokemon-silenced cells, the FAS receptor, FADD and their downstream targets caspase-10 and caspase-8 were activated, causing increased release of caspase-8 active fragments p18 and p10. Increased activated caspase-8-mediated cleavage and activation of downstream effector caspases such as caspase-9 and caspase-3 was observed in HepG2 si-Pokemon cells as compared to control. Therefore, Pokemon might serve as an important mediator of crosstalk between intrinsic and extrinsic apoptotic pathways in HCC cells. Moreover, our findings suggest that Pokemon could be an attractive therapeutic target gene for human cancer therapy.

## Introduction

Hepatocellular carcinoma (HCC) is one of the most common human malignancies worldwide and is the third leading cause of cancer deaths. The development of hepatocellular carcinoma is associated with an imbalance of proliferation and apoptosis molecularly governed by various oncogenes, tumor-suppressor genes and growth factor genes, such as p53 and retinoblastoma (Rb) [1]. Fas-associated death domain (FADD) regulates cellular apoptosis in HCC, with loss of FADD expression playing an important role in HCC carcinogenesis [2].

Pokemon (POK erythroid myeloid ontogenic actor), also known as FBI-1, LRF and OCZF, has recently been identified as a POK transcription factor with proto-oncogenic activity. It consists of an NH2-terminal POZ/BTB domain and COOH-terminal kruppel-type zinc finger domain [3], [4]. Our previous study demonstrated that Pokemon is overexpressed in HCC and promotes HCC cell proliferation and migration via an AKT- and ERK -dependent manner [5]. Maeda et al have shown that Pokemon can inhibit transcription of p14ARF and subsequently reactivate Mdm2, which reduces p53 expression [6]. Another study demonstrated that Pokemon can regulate cell-cycle progression by repressing Rb and p21 and that its activity is mediated by direct binding competition with the Sp1/3 GC-box [7], [8]. In addition, Pokemon enhances NF-κB mediated transcription by interacting with the Rel homology domain [9]. However, few studies have assessed the role of Pokemon in apoptosis in HCC.

Classical apoptosis can be initiated via two major pathways: the intrinsic or mitochondria-mediated pathway and the extrinsic or death receptor-mediated pathway. Activation of both pathways results in the activation of caspases. Chemotherapy drugs that reengage normal apoptotic pathways have the potential to effectively treat cancers. Agents that specifically target apoptotic machinery including tumor necrosis factor (TNF)-related apoptosis-inducing ligand (TRAIL) receptors, the BCL2 family of anti-apoptotic proteins, inhibitor of apoptosis (IAP) and MDM2 are currently being explored for cancer drug discovery [10].

Oxaliplatin, a third-generation platinum-based chemotherapeutic agent, displays a broader spectrum of antitumor activity than cisplatin and carboplatin. Several oxaliplatin-combined regimens have been used to treat patients with advanced HCC, and induce apoptosis via activation of the p53-caspase 8 pathway in HepG2 cells [11], [12]. Several studies have identified some chemotherapy drugs that induce apoptosis of HCC through the Fas receptor or mitochondrial pathway [13], [14]. Activation of TRAIL leads to the recruitment of FADD and activation of caspase 8, which can further amplify the death signal by activating the mitochondrial apoptotic pathway through cleavage of BID. Cleaved BID binds to BAX or BAK and causes the release of cytochrome c, which can result in the activation of caspase 9 and other downstream caspases [15]. However, the exact mechanism underlying these synergistic actions remains unclear. In this study, we will determine how Pokemon participates in the development of HCC by regulating Fas and mitochondria-mediated apoptotic pathways.

## Materials and Methods

### Cell Lines and Reagents

HepG2 and SMMC-7721 HCC cell line were grown in DMEM (GIBCO) and 1640 (GIBCO) supplemented with 10% fetal bovine serum (GIBCO) and 100 IU/ml penicillin-streptomycin. HepG2 was purchased from ATCC (American Type Culture Collection, United States), and SMMC-7721 was provided by the Cell Bank of Shanghai Institute of Cell Biology (Chinese Academy of Sciences, Shanghai, China.

### Establishment of Stable Pokemon-knockdown Cell Lines

A plasmid encoding a short interfering RNA (siRNA) targeting Pokemon was constructed as previously described [16]. Stable knockdown cells were established as previously described [17]. Sequences for si-Pokemon were selected using an algorithm as described in our previous study: forward primer, 5′-CACCAGTAGAATGTGTACGGGATACGTGTGCTGTCCGTATCTCGTCACGTTCTGCTTTTTT-3′; reverse primer, 5′- GCATAAAAAGCAGAACGTGTACGAG ATACGGCAGCAACGTATCCCGTACACATTCTACT-3′.

Both HepG2 and SMMC-7721 cells were transfected with the si-Pokemon (pcPUR+U6-si-Pokemon) or PU6 (pcPUR+U6-siRenilla) plasmid using the QIAGEN transfection reagent. Puromycin (2 mg/ml) was used to select stably transfected clones. Pokemon expression was examined by Western blotting analysis using an antibody against Pokemon to validate construct efficiency for inhibiting target gene expression. Experiments were repeated three times. Stably transfected cell lines exhibiting effective down-regulation of the Pokemon gene were named HepG2 si-Pokemon and 7721 si-Pokemon. Cell lines stably transfected with control plasmid were named HepG2-Pu6 and 7721-Pu6.

### TUNEL Analysis of Cell Apoptosis

TdT-UTP nick end labeling (TUNEL) assays were performed with the one-step TUNEL kit according to the manufacturer’s instructions. Cells grown in 6-well culture clusters were treated with different concentrations of oxaliplatin for 24 hours. Treated cells were fixed onto poly-(L-lysine)-coated slides with 4% paraformaldehyde/PBS. Slides were rinsed with PBS, and cells were then permeabilized with 0.1% Triton X-100. Slides were washed with PBS, and cells were incubated in 50 µl of TUNEL reaction mixture for 1 h at 37°C in the dark. Next, 50 µl of DAPI was added and incubated for 2 min at room temperature. Cells were imaged by ﬂuorescent microscopy using 488 nm excitation and 530 nm emission. Cells exhibiting green ﬂuorescence were defined as TUNEL positive, apoptotic cells.

### FCM Analysis of Cell Apoptosis

HepG2 si-Pokemon, HepG2-Pu6, and SMMC-7721 si-Pokemon 7721-Pu6 cells were treated with different concentration of oxaliplatin for 24 h. Next, cells were stained with FITC-conjugated Annexin V and propidium iodide (PI) as supplied by an apoptosis detection kit (KeyGEN, China). Cells were analyzed using a FACS Calibur ﬂow cytometer (San Jose, CA, USA). Summit software (FlowJo, USA) was used to determine the number of apoptotic cells.

### Apoptosis-related Protein Array

The Human Apoptosis Array (ARY009, R&D Systems) was used to detect relative expression levels of 35 apoptosis-related proteins in HepG2 si-Pokemon or control cells treated with 20 µg/ml oxaliplatin for 24 hours. Experiments were performed according to the manufacturer’s instructions.

### Western Blot Analysis

Total protein was extracted from cells and tissue specimens using the Mammalian Cell Lysis Reagent (Fermentas) according to the manufacturer’s protocol. Proteins were resolved using 10%, 12% or 15% sodium dodecyl sulfate polyacrylamide gel electrophoresis and analyzed using appropriate antibodies.

### FCM Analysis of Cell Cycle

For cell cycle analysis, HepG2 si-Pokemon, SMMC-7721 si-Pokemon and control cells were labeled with 100 mg/ml propidium iodide solution (Sigma) containing 100 mg/ml RNase A according to the manufacturer’s instructions (KeyGEN Biotech). Samples were subjected to FACS analysis, and the data were analyzed using ModFit LT v.2.0.

### RNA Extraction and qPCR

Total RNA was extracted from cells using TRIzol reagent (Invitrogen, United States) according to the manufacturer’s instructions. RNA samples were dissolved in nuclease-free water, and the concentration was determined by measuring absorbance at 260 and 280 nm. The reverse transcription (RT) reaction for first-strand cDNA synthesis was performed with 2 mg of total RNA using reverse transcriptase (Fermentas). Primer sequences for Pokemon, cell cycle related and apoptosis-related genes (synthesized by BGI) are listed in Table S1. Quantitative (q) PCR was performed using the SYBR Green Master Mix (Thermo Scientific, Rockford, IL). Then, qPCR was performed using Rotor-Gene RG-6000A apparatus (Corbett Research Cambridge, UK). Three independent experiments were performed and triplicate with each reaction, and data was normalized by GAPDH gene expression values.

### Statistical Analysis

Data are presented as mean ± SD unless otherwise indicated. Differences were considered significant when P-values, as determined by the Student’s t-test, were less than 0.05.

## Results

### Silencing of Pokemon Enhances Apoptosis in HCC Cells

**Figure 1:**
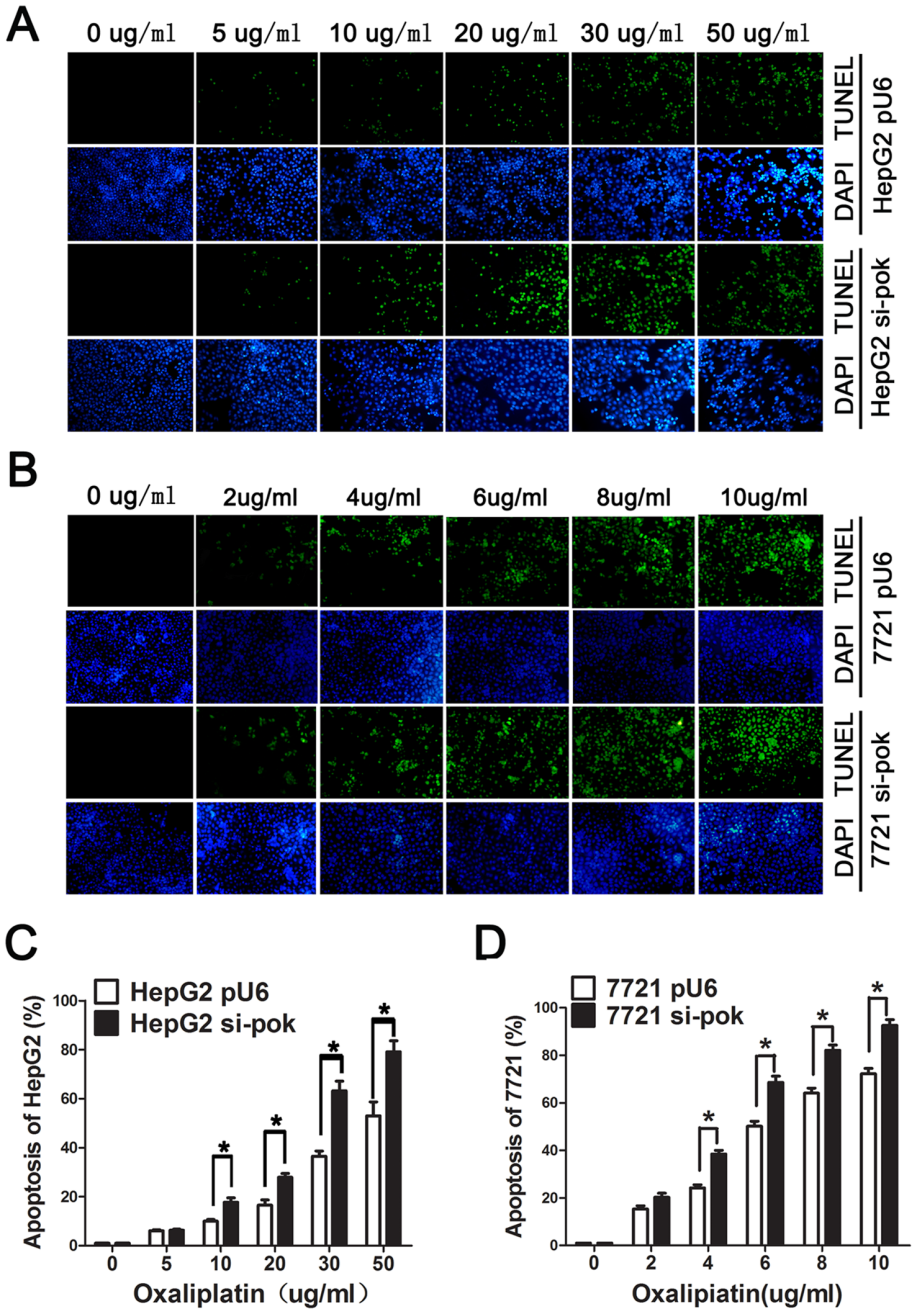
TUNEL analyses reveals enhanced apoptosis in HCC cells with silencing of Pokemon. (A and B) HepG2-pu6, HepG2 si-Pokemon, 7721-pu6 and 7721 si-Pokemon cells were treated with different concentration of oxaliplatin for 24 hours. The TUNEL assay was performed using the One-Step TUNEL Apoptosis Assay Kit. Images were captured by fluorescence microscopy. The green color is indicative of TUNEL-positive cells, and the blue color marks the presence of all cells. (C and D) The percentage of apoptotic cells is reported. Data are presented as the mean ± SD of three independent experiments. *P<0.05.

Cells undergoing apoptosis generate DNA fragments through the action of endogenous endonucleases. HepG2 si-Pokemon and HepG2-Pu6 cells were treated with various concentration of oxaliplatin ranging from 0 to 50 µg/ml for 24 hr, and the resulting fragmented DNA of apoptotic cells were labeled using the TUNEL assay. Increasing concentrations of oxaliplatin significantly increased the number of apoptotic cells. This effect was greater in Pokemon-silenced cells than control cells (Fig. 1A and C). As shown in Fig. 1B and D, the effect of Pokemon on inhibition of apoptosis was also observed in SMMC-7721 cells.

**Figure 2:**
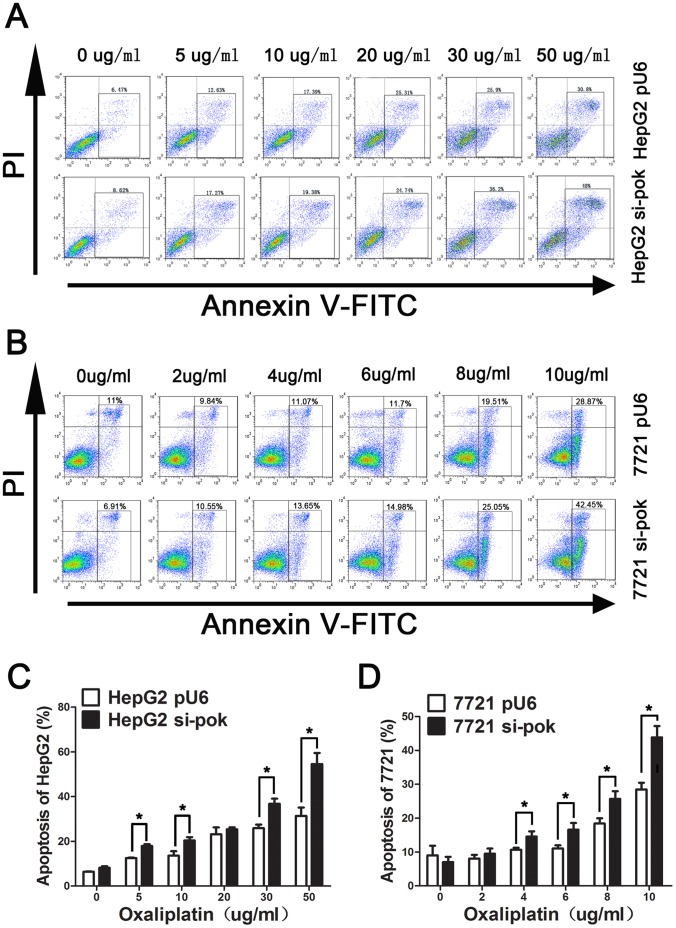
Silencing of Pokemon enhances apoptosis in HCC cells as assessed by Flow cytometry. HepG2-pu6, HepG2 si-Pokemon, 7721-pu6 and 7721si-Pokemon cells were treated with different concentration of oxaliplatin for 24 hours. (A and B) Cells were stained with Annexin V-FITC/PI, and apoptosis was analyzed by flow cytometry. (C and D) The percentage of apoptotic cells is reported. Data are presented as the mean ± SD of two independent experiments. *P<0.05.

To further confirm that silencing Pokemon promotes apoptosis, we analyzed cells treated with increasing concentrations of oxaliplatin for 24 hours by FACS. The number of apoptotic cells increases by 54.5±4.95% and 43.83±3.95%, respectively, in HepG2 si-Pokemon and 7721 si-Pokemon cells treated with oxaliplatin. In HepG2-Pu6 and 7721-Pu6 control cells, the increase in cells undergoing apoptosis was only 31.4±3.67% and 28.48±1.96%, respectively (Fig. 2B and D).This finding shows that Pokemon silencing enhances apoptosis in HCC cells.

### Silencing of Pokemon Promotes Expression of Apoptosis-related Proteins

**Figure 3:**
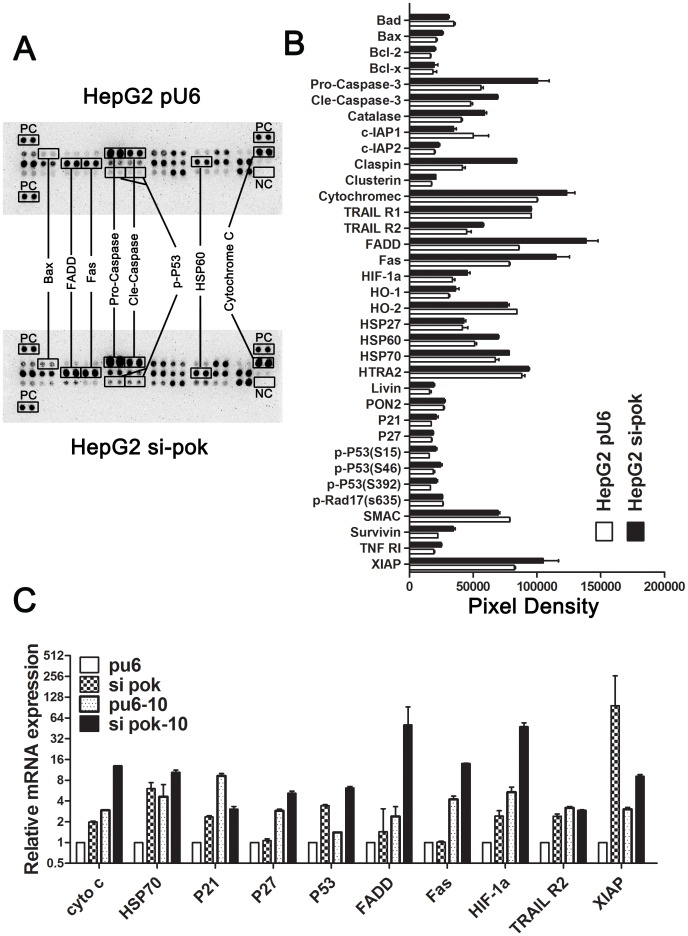
Human Apoptosis Array and RT_PCR analyses. (A) HepG2-pu6 and HepG2 si-Pokemon cells were treated with 10 µg/ml oxaliplatin for 24 hours and subjected to array analysis. (B) Pixel densities of apoptosis-related proteins identified from array analysis of HepG2-pu6 and HepG2 si-Pokemon cells. *P<0.05.(C) RT-PCR analysis shows mRNA levels of apoptosis-related genes in HepG2 cells with and without 10 µg/ml oxaliplatin treatment for 24 hours.

The above results suggest that silencing of Pokemon induces apoptosis; therefore, we wanted to investigate the molecular mechanism by which this occurs. Protein from cells treated with 10 µg/ml oxaliplatin was examined using the human apoptosis array (Fig. 3A). Numerous intrinsic and extrinsic pathway pro-apoptotic proteins were up-regulated, including the following: Fas, FADD, Bax, caspase-3, HIF-1a, HSP70, p27, p53 and cytochrome c (Fig. 3A and B). Quantitative PCR revealed increased protein expression after Pokemon silencing that was further increased upon drug treatment (Fig. 3C and Fig. S1).

### Silencing of Pokemon Increases p53 Expression

**Figure 4:**
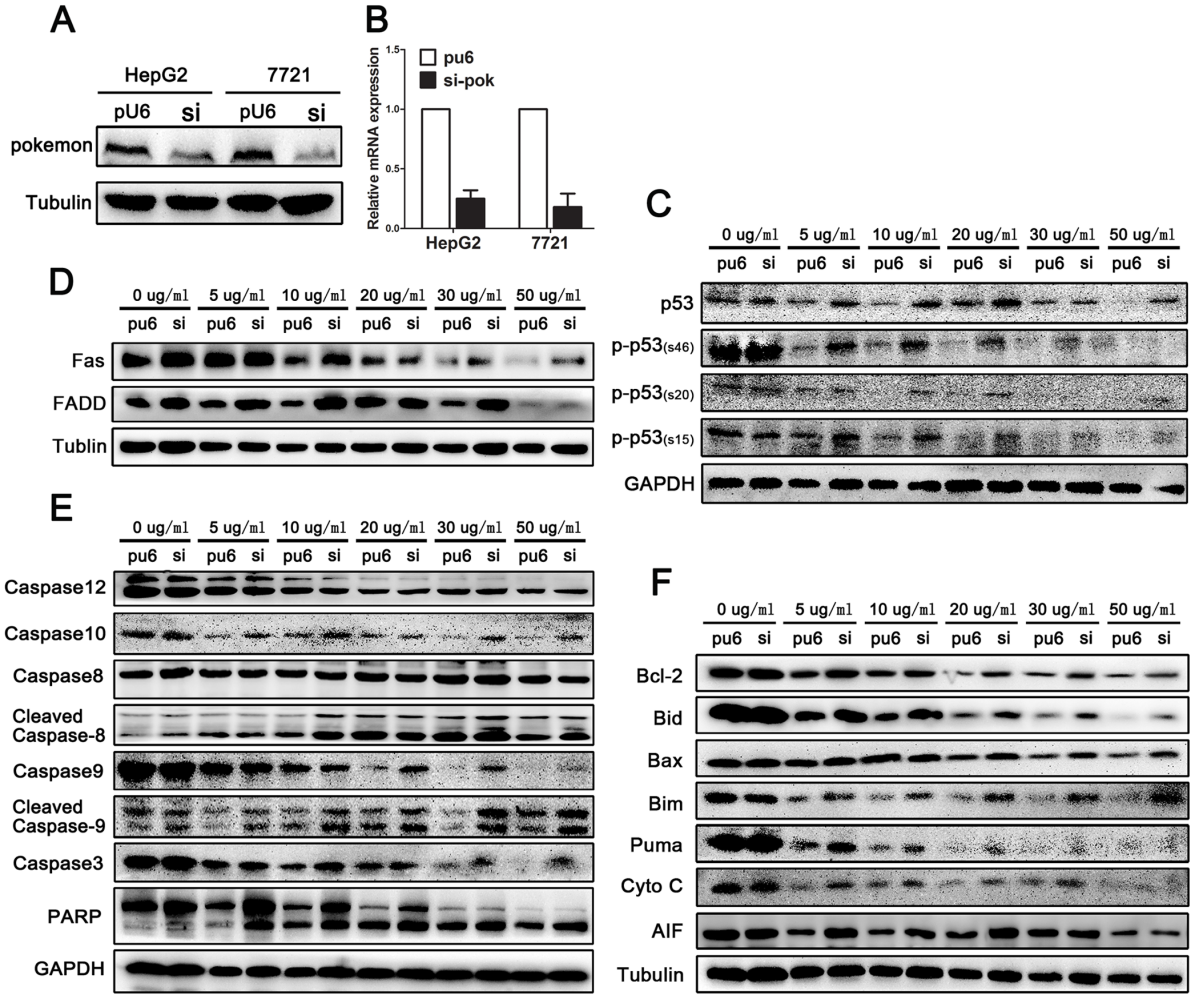
The effect of Pokemon on Fas- and mitochondria-mediated caspase-dependent apoptosis as shown by Western blot analysis after treatment with various concentration of oxaliplatin for 24 h. (A and B) HepG2 and SMMC7721 cells were stably transfected with Pokemon siRNA or Pu6 vector. Western blot and qRT-PCR analyses confirm siRNA-mediated silencing of Pokemon. (C) The Western blot shows the effect of Pokemon on total and phosphorylated p53. (D) Expression of Fas and FADD were up-regulated as a result of silencing Pokemon. (E)Western blot analyses show the effect of silencing Pokemon on caspase family proteins and the main cleavage targets of caspase-9, caspase-8 and PARP. (F) Western blots analyses of pro-apoptotic and pro-survival Bcl-2 family proteins and mitochondrial release of cytochrome c and AIF.

Fragmented DNA, a hallmark of apoptosis identified by the TUNEL assay, was increased in Pokemon silenced cells. The p53 tumor suppressor protein plays a major role in the cellular response to DNA damage and other genomic aberrations. Activation of p53 can lead to either DNA repair or apoptosis. Our data showed no change in p53 expression or Ser15, Ser20 and Ser46 phosphorylation at baseline. However, p53 and its phosphorylation were significantly increased after treatment with different concentrations of oxaliplatin, with maximal effects observed with 5 µg/ml and 10 µg/ml oxaliplatin (Fig. 3B and 4C).

### Silencing of Pokemon Initiates Death Receptor-mediated Apoptosis

Apoptosis can be initiated via the extrinsic or death receptor-mediated pathway. In this pathway, the Fas receptor and its protein complex FADD interacts with the amino-terminal death effector domain to activate the caspase cascade. Our data indicate that Fas and FADD expression were increased in Pokemon-silenced cells after treatment with oxaliplatin (Fig. 2A and Fig. 4D). Moreover, caspase-10 and caspase-8, downstream targets of Fas and FADD, were activated and promoted release of the caspase-8 active fragments p18 and p10 (Fig. 4E). Activated caspase-8 cleaves and activates downstream effector caspases such as caspase-9 and caspase-3. Both caspase-9 and caspase-3 were up-regulated in HepG2 si-Pokemon cells as compared to the control. PARP, a primary caspase-3 cleavage target that serves as an apoptosis marker, was increased in HepG2 si-Pokemon cells (Fig. 4E).

### Silencing of Pokemon Alters the Expression Bcl-2 Family Proteins

Consider the following sentence: “Given that chemotherapy drugs can initiate apoptotic pathways including the mitochondria-mediated pathway, these drugs have the potential to effectively treat cancer. Numerous pro-apoptotic proteins were up-regulated in the Apoptosis Antibody Array (Fig. 3A and B), and therefore, we wanted to examine the expression of Bcl-2, Bax, Bid, Bim, Puma, cytochrome c and AIF by Western blotting. The Expression of pro-apoptotic Bcl-2 family members including Bad, Bid, Bim and Puma was increased in HepG2 si-Pokemon cells. AIF and cytochrome c, normally localized to the mitochondrial intermembrane space and released in response to apoptotic stimuli, were also up-regulated in HepG2 si-Pokemon cells (Fig. 4F). However, the expression of Bcl-2 was increased in Pokemon silenced HepG2 cells (Fig. 4F).

## Discussion

The transcription factor Pokemon has previously been identified as a regulator of the important tumor suppressor ARF, and cells lacking Pokemon have proven refractory to malignant transformation [18]. Subsequent investigation revealed that Pokemon conducts various cellular regulatory functions, such as modulation of HIV-1 transcription, nuclear sequestration of NF-κB, transcriptional repression of the ADH5/FDH gene, adipocyte differentiation and osteoclastogenesis [19], [20], [21]. Recently, many studies have confirmed that Pokemon is an important proto-oncogene deregulated in many cancers [22]. In our previous study, Pokemon is overexpressed in HCC and promotes HCC cell proliferation [5]. In this study, we used siRNA-mediated silencing of Pokemon in HepG2 and SMMC-7721 cells (Fig. 4A and B) to elucidate Pokemon’s function in HCC. Using the TUNEL assay, we found that increasing the concentration of oxaliplatin significantly increases the number of apoptotic cells [23]. This observation was more dramatic in cells with defective Pokemon as compared to control cells. The function of silencing Pokemon in promoting apoptosis was further confirmed by FACS analysis.

Based upon these findings, we investigated the molecular mechanism for Pokemon-mediated enhanced HCC apoptosis using the Apoptosis Antibody Array. Our data revealed up-regulation of proteins involved in intrinsic and extrinsic apoptotic signaling pathways such as Bax, cytochrome c, Fas, FADD, caspase-3, HIF-1a and p53. The p53 tumor suppressor protein is one of the most studied proteins because of its status as “guardian of the genome” [24]. It constitutes the central node in a network of molecular interactions regulating the cellular response to stresses such as DNA damage and oncogene activation. Avery-Kieida et al reported that some p53 target genes involved in apoptosis and cell cycle arrest (at G1 and/or G2 phase) are aberrantly expressed in melanoma cells, leading to abnormal p53 activity and contributing to the proliferation and apoptosis of these cells [25]. We found increased expression of checkpoint-related genes including ATM, BRCA1, BRCA2, CDKN1A, CHEK1, CUL1, CDKN2A and CDKN2B in HepG2 si-Pokemon cells. Altogether, this expression pattern resulted in cell cycle arrest at S phase (Figure S1). High p53 expression induces apoptosis in fetal liver erythroblasts, and erythroid-specific inactivation of the mouse Mdm2 gene, a key negative regulator of p53, leads to increased apoptosis in erythroblasts and embryonic lethality due to a severe anemia (Mdm^2lox/lox^EpoR^cre+^) [26]. p53 is also able to promote apoptosis through transcription-independent apoptotic mechanisms. Choi et al found that Pokemon acts as a master control of cellular transformation and proliferation by potently blocking the p53 pathway in HeLa cells. Our findings suggest that p53 expression and phosphorylation were significantly increased after treatment of oxaliplatin. These data suggest that Pokemon silencing enhancing apoptosis of HCC cells via the p53 pathway.

p53 is involved in apoptotic induction through two apoptotic signaling pathways (mitochondria-mediated and death receptor-mediated) thought to be distinct until recently. The intrinsic, mitochondrial apoptotic pathway is regulated by the Bcl-2 family of proteins that govern the release of cytochrome c from the mitochondria [27]. Bcl-2 family proteins are classified as pro-apoptotic (Bax, Bak, Bad, Bid, Bik and Bim) or anti-apoptotic (Bcl-2, Bcl-XL and Mcl-1 ) [28]. Pro-apoptotic proteins promote release of cytochrome c from the mitochondria, initiating the apoptotic cascade. Cytochrome c activates caspase-9, which cleaves and activates downstream effector proteases, such as caspase-3, leading to apoptosis [29]. Once activated, caspase-3 cleaves PARP into two fragments, p89 and p24, promoting DNA fragmentation and triggering apoptosis [30]. Apoptosis inducing factor (AIF) and second mitochondria-derived activator of caspase (SMAC) are additional apoptotic factors released from the mitochondrial intermembrane space into the cytoplasm [31]. Our data show increased expression of pro-apoptotic Bcl-2 family proteins (Bad, Bid, Bim and Puma), AIF and cytochrome c in HepG2 si-Pokemon cells. Unexpectedly, the expression of Bcl-2 was increased in Pokemon silenced HepG2 cells. However, It has been reported that the ratio of Bax to Bcl-2, rather than Bcl-2 alone, is important for survival of drug-induced apoptosis in cancer cells [32].

The extrinsic pathway is mediated by death receptors. The majority of HCC cell lines possess at least one genetic alteration in Fas pathway molecules, which inhibit Fas-mediated apoptosis [33]. For example, Fas ligand interacts with the Fas receptor, causing caspase-8 and caspase-10 activation [34]. Engagement of mFas via the Fas-associated death domain protein (FADD) is necessary for activation of caspase-8) [35]. Active caspase-8 and caspase-10 directly cleave and activate downstream effector proteases, such as caspase-3, causing apoptosis [36]. The present study showed that the expression of the receptor Fas and FADD and the downstream protein of caspase-10 and caspase-8 were activated and led to the release of the caspase-8 active fragments, p18 and p10, which had increased expression in Pokemon-silenced cells after treatment with oxaliplatin. Activated caspase-8 cleaves and activates downstream effector caspases, such as caspase-9 and caspase-3, which were up-regulated in the HepG2 si-Pokemon cells compared to the controls. In addition, caspase-8 and caspase-10 have the ability to cleave the Bcl-2 family member Bid into truncated Bid (tBid), thereby resulting in disruption and release of cytochrome c [37], [38]. Therefore, Pokemon might be a critical mediator of crosstalk between the intrinsic and extrinsic apoptotic pathways in HCC cells.

Altogether, our findings suggest that Pokemon could be an attractive therapeutic target for human cancer therapy in light of its essential role in HCC cells.

## Supporting Information

Figure S1 **Silencing Pokemon induces cell cycle arrest and up-regulation of cell cycle checkpoints in HepG2 cells as shown by flow cytometry and RT-PCR analyses.** (A)HepG2-pu6 and HepG2 si-Pokemon cells were stained with propidium iodide (PI), and the cell cycle distribution was analyzed by flow cytometry. (B) The percentage of cell cycle distribution is shown. (C) mRNA levels of cell cycle checkpoint genes analyzed by RT-PCR in HepG2 cells treated with and without oxaliplatin 10 µg/ml oxaliplatin for 24 hours. *P<0.05.
(TIF)

Table S1 Quantitative PCR Primer Sequences.
(DOCX)
