# Supplementary figures and images for: Silencing of Pokemon Enhances Caspase-Dependent Apoptosis via Fas- and Mitochondria-Mediated Pathways in Hepatocellular Carcinoma Cells
Source: PLoS One. 2013 Jul 17;8(7):e68981. doi: 10.1371/journal.pone.0068981 (PMC3714264; doi:10.1371/journal.pone.0068981)

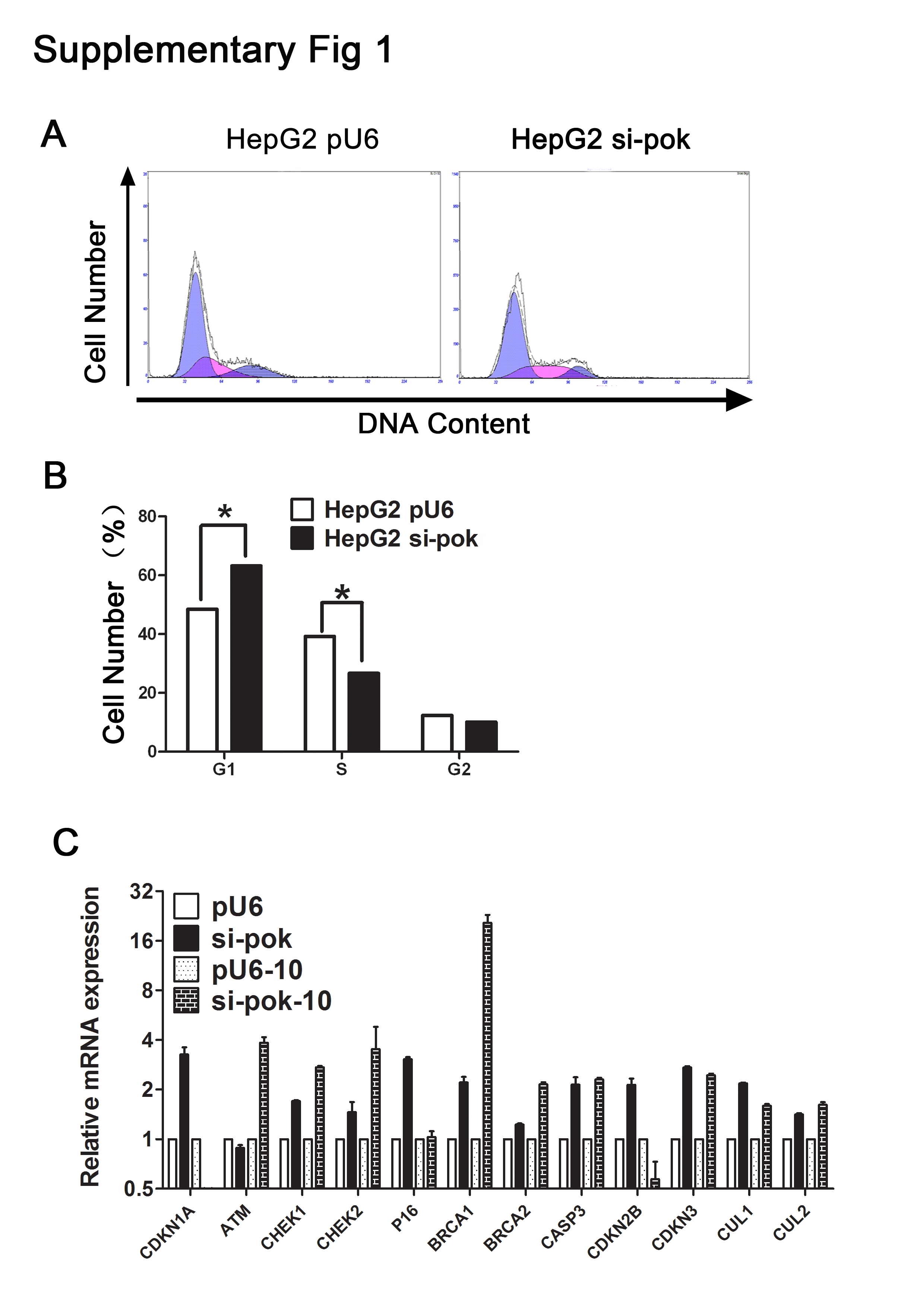

Supplement: Figure S1 — Silencing Pokemon induces cell cycle arrest and up-regulation of cell cycle checkpoints in HepG2 cells as shown by flow cytometry and RT-PCR analyses. (A)HepG2-pu6 and HepG2 si-Pokemon cells were stained with propidium iodide (PI), and the cell cycle distribution was analyzed by flow cytometry. (B) The percentage of cell cycle distribution is shown. (C) mRNA levels of cell cycle checkpoint genes analyzed by RT-PCR in HepG2 cells treated with and without oxaliplatin 10 µg/ml oxaliplatin for 24 hours. *P<0.05. (TIF) [file pone.0068981.s001.tif]
