# Supplementary material for: Silencing of Pokemon Enhances Caspase-Dependent Apoptosis via Fas- and Mitochondria-Mediated Pathways in Hepatocellular Carcinoma Cells
Source: PLoS One. 2013 Jul 17;8(7):e68981. doi: 10.1371/journal.pone.0068981 (PMC3714264; doi:10.1371/journal.pone.0068981)
Supplement: Table S1 — Quantitative PCR Primer Sequences. (DOCX) [file pone.0068981.s002.docx]

**Supplementary table 1.** Quantitative PCR Primer Sequences.

| Gene | Forward primer sequence (5’→3’) | Reverse primer sequence (5’→3’) |
| --- | --- | --- |
| Pokemon | GGGGACAGCGACGAGGAG | CGTAGTTGTGGGCAAAGG |
| GAPDH | GTCTCCTCTGACTTCAACAGCG | ACCACCCTGTTGCTGTAGCCAA |
| CHEK1 | GCCAAGCCAAAGTCAGAGAT | GGGGTGGTTTATCTTCATGG |
| MAD2L1 | CACTGTTGGAAGTTTCTTGTTCA | ACTGTGGTCCCGACTCTT |
| CDKN3 | TCACCCATCATCATCCAATCGC | CTCGCAGGCTGTCTATGGCTTG |
| Cdc-34 | GCGGCGCCTTTAATTGG | GCTGTCCGACGGATCAGTCT |
| P16 | CCCCTTGCCTGGAAAGATAC | AGCCCCTCCTCTTTCTTCCT |
| ATM | GATCTTGTGCCTTGGCTACAGATTG | ATGTCGCTGTTGGGGTAGAAGCTGAG |
| BRCA1 | CTAGGTACCTTGGGAGGGGGCTCGGGCATGGC | CATAAGCTTCCAGGAAGTCTCAGCGAGCTCACG |
| BRCA2 | GTACAGGAAACAAGCTTCTGA | GACTAACAGGTGGAGGTAAAG |
| CASP3 | CTGGAA AACCCAAACTTTTCATTA | GCCAGGAAA AGTAACCAGGTGC |
| CDKN1A | CTGCGCCAGCTGAGGTGTGA | GCTGCTCGCTGTCCACTGGG |
| CDC25C | GTGCATTTAGCTGGGATGACAATGGAA | GGCCACTTCTGCTCACCTTTGC |
| CDKN2B | TCTCCGTTGGCCGGAGGTCA | TGGCAGGGTCTGCGCAGTTG |
| CDC25A | CGTGGCTGCCTGCACTCTCA | GGCTGTCACAGGTGACTGGGG |
| CCNA2 | AAAAGGCAGCGCCCGTCCAA | CTGCTGCTGCGCTAGACCCC |
| CDK1 | ACTGGCTGATTTTGGCCTTGCC | TGAGTAACGAGCTGACCCCAGCAA |
| CYCS | CAACTTTTCACAAAGATGGTGAGTG | GAGGCAAATGAACATGAACACAA |
| TRAIL R2 | CGCTGCACCAGGTGTGATT | GTGCCGGCTTCGCACTGACA |
| FADD | CCTAGACCTCTTCTCCATGCT | AATGCTGCACACAGGTCTTCTT |
| Fas | CACTATTGCTGGAGTCATG | CTGAGTCACTAGTAATGTCC |
| HIF-1α | CTCTGAG CTCTATCTGGAAGGTATGTG | CCTCAAGCTTCAGTTAGTGTTAGACCC |
| HSP70 | GATTTGCTACTGCTGGATG | AGGAGGGATACCTGTGAG |
| XIAP | AACCTTGTGATCGTGCCT | ACCCTGGATACCATTTAGC |
| p21 | GACTCTCAGGGTCGAAAACG | GGATTAGGGCTTCCTCTTGG |
| p27 | AGGCTGGGTTAGCGGAGC | GAACCGTCTGAAACATTTTCTTCTGT |
| p53 | TTTCCGTCTGGGCTTCT | TGGAATCAACCCACAGCT |
